# Challenges for identifying the neural mechanisms that support spatial navigation: the impact of spatial scale

**DOI:** 10.3389/fnhum.2014.00571

**Published:** 2014-08-04

**Authors:** Thomas Wolbers, Jan M. Wiener

**Affiliations:** ^1^German Center for Neurodegenerative Diseases (DZNE), and Center for Behavioural Brain Sciences (CBBS), Otto-von-Guericke UniversityMagdeburg, Germany; ^2^Department of Psychology, Faculty of Science and Technology, Bournemouth UniversityBournemouth, UK

**Keywords:** spatial navigation, hippocampus, vista space, environmental space, reference frames, allocentric and egocentric representation

## Abstract

Spatial navigation is a fascinating behavior that is essential for our everyday lives. It involves nearly all sensory systems, it requires numerous parallel computations, and it engages multiple memory systems. One of the key problems in this field pertains to the question of reference frames: spatial information such as direction or distance can be coded egocentrically—relative to an observer—or allocentrically—in a reference frame independent of the observer. While many studies have associated striatal and parietal circuits with egocentric coding and entorhinal/hippocampal circuits with allocentric coding, this strict dissociation is not in line with a growing body of experimental data. In this review, we discuss some of the problems that can arise when studying the neural mechanisms that are presumed to support different spatial reference frames. We argue that the scale of space in which a navigation task takes place plays a crucial role in determining the processes that are being recruited. This has important implications, particularly for the inferences that can be made from animal studies in small scale space about the neural mechanisms supporting human spatial navigation in large (environmental) spaces. Furthermore, we argue that many of the commonly used tasks to study spatial navigation and the underlying neuronal mechanisms involve different types of reference frames, which can complicate the interpretation of neurophysiological data.

## Introduction

A central issue in the cognitive neuroscience of spatial navigation pertains to the question of reference frames. Ever since the discovery of place cells in the rodent hippocampus (O’Keefe and Dostrovsky, 1971), this structure has been thought to provide an allocentric description of the environment. In contrast, cortical regions such as posterior parietal cortex or the striatum have been linked to processing spatial information in various egocentric reference frames. Even though the terms “egocentric” and “allocentric” appear frequently in manuscripts on spatial navigation, their precise meaning is often not described. In addition, different authors have different interpretations, which can lead to confusion about what exactly is meant by an allocentric neural code, for example.

In this review, our aim is to highlight some of the problems that can arise when studying the neural mechanisms that are presumed to support different spatial reference frames. In particular, we show that many of the commonly used tasks involve different types of reference frames, which can complicate the interpretation of neurophysiological data gathered in such situations. In addition, we discuss how the scale of space in which a navigation task takes place determines the processes that are being recruited, which has important implications, for example, for the inferences that can be made from animal studies about the neural mechanisms supporting human spatial navigation.

## Background

### Egocentric vs. allocentric reference system

**Figure 1:**
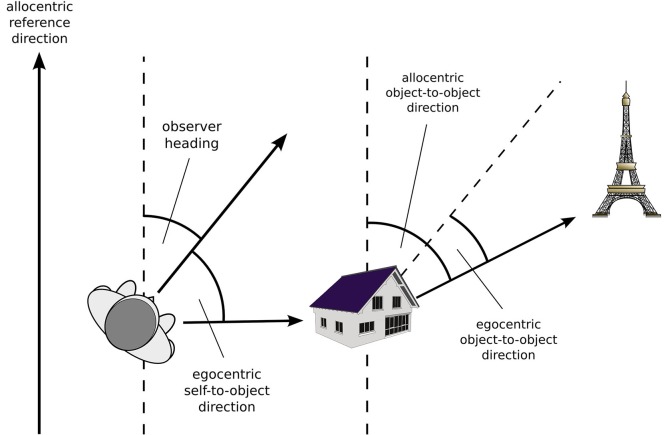
**Egocentric and allocentric relationships**.

To begin, we will provide a definition of what characterizes an egocentric vs. an allocentric spatial code. Exceptions notwithstanding, there is general agreement that in an egocentric reference frame, locations are represented with respect to the particular perspective of an observer. As shown in Figure 1, the origin of the egocentric reference system is centered on the observer, and its *orientation is defined by the observer’s heading*. Importantly, the brain entertains multiple egocentric reference frames that are anchored to different body parts (i.e., eye-centered, head-centered, trunk-centered), hence the orientation of an egocentric reference system is determined by the orientation of the specific body part.

For the sake of simplicity, let us assume that all body parts are orientated in the same direction, hence the observer’s heading coincides with the orientation of all egocentric reference frames. Assuming a polar coordinate system, the egocentric position of an external object can now be specified as follows: the length of the vector connecting the observer and the object is the *egocentric distance* of the object, and the angle between the observer’s heading and that vector specifies its *egocentric direction*. However, egocentric direction can also specify the direction between two external objects, in this case it refers to the angle between the direction of the observer’s heading and the vector connecting the two objects.

In contrast to observer dependent egocentric reference systems, an allocentric reference frame specifies an object’s position within a framework external to the observer and independent of its position and orientation. Assuming a polar coordinate system, an object’s allocentric distance corresponds to the length of a vector connecting the origin of the coordinate system and the object, and the angle between the allocentric reference direction and that vector specifies its allocentric direction. However, most environments do not provide a meaningful reference point that could serve as an unambiguous origin for an allocentric coordinate system. As a consequence, *allocentric distance* of an object is rarely defined with respect to an origin but rather with respect to other, behaviorally relevant objects in the environment. Similarly, *allocentric direction* usually specifies the direction between two external objects, defined as the angle between an allocentric reference direction and the vector connecting the two objects (Figure 1).

From these definitions, it is obvious that one major difference between egocentric and allocentric representations pertains to what happens when the observer moves about in the environment. For example, if the observer simply turns around, an object’s egocentric direction changes, but its egocentric distance remains the same. In contrast, neither allocentric distance nor directions are affected by observer rotation. The differences are even more pronounced for translational movements, for which both egocentric distance and direction change, whereas their allocentric counterparts remain unaffected. It is this observer independence that makes allocentric representations an attractive format for long-term memory representations: when the observer moves about in a familiar environment, planning any navigational step from an egocentric representation would require that one continuously updates the egocentric vectors towards all potential goals. In contrast, in an allocentric representation, all the observer needs to do is to update their own allocentric position, because this knowledge allows for computing egocentric distance and direction towards other objects from any vantage point.

There is, however, one major problem that is rarely addressed in the literature: what does an allocentric reference system actually look like? While an egocentric reference system can be easily defined in any situation where an observer is present, this is much harder for allocentric reference frames. Imagine, for example, an observer walking around on famous St. Peter’s Square in Rome, which has an oval shape. In this situation, it is not at all obvious what would define the orientation of an allocentric reference system. Moreover, while the observer might try to extract the major axes of the square and use those to define orientation, the situation becomes even more ambiguous when the observer leaves the square and strolls around one of the neighboring streets whose orientation changes as one walks along. Is the initial orientation of the allocentric representation still used or is a new one adopted? And what factors determine whether or not a switch to a new direction occurs?

### Scales of space

**Figure 2:**
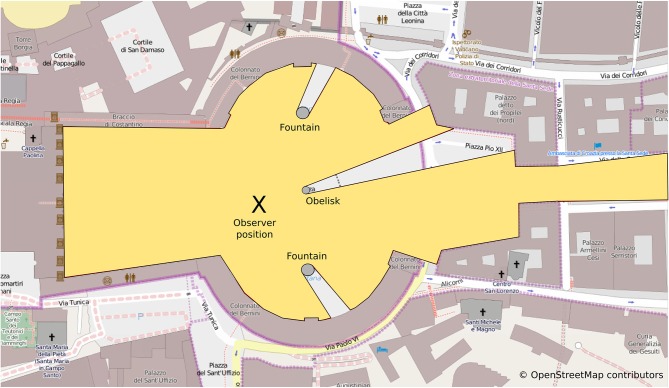
**Vista spaces: the yellow polygon despicts the area of St Peters square that is visible from the observer position (x)**. Note that almost the entire space can be apprehended from a single position. While visual barrieres such as the obelisk and the fountains will obstruct the view of some of the space, only little exploratory movements are required to apprehend the entire space. The visible area from any position is crucial for defining the local vista spaces as well as connections between them (see Franz and Wiener, 2008).

When considering the kind of reference frames associated with different navigation tasks or paradigms it is important to consider the scale of space in which these tasks take place. Montello distinguishes between four classes of psychological spaces: figural, vista, environmental and geographical space (Montello, 1993). While figural space (e.g., the space of pictures or small objects) and geographical space (e.g., the space of countries or nations) are too small or too large to be experienced through navigation, the distinction between vista and environmental space is relevant in the context of navigation: a vista space is the space that can be visually apprehended from a single location or with only little exploratory movements (see Figure 2). Typical examples for vista spaces are single rooms or town squares such as the St. Peters Square in Rome mentioned above. In contrast, environmental spaces such as buildings, neighborhoods or towns cannot be experienced from a single place or even from a certain part of the environment but require considerable movement. Although navigation paradigms that aim to investigate spatial reference frames and the underlying neuronal representations have made use of both vista and environmental spaces, the impact that different spatial scales have on the ensuing spatial representation and their reference frame are rarely considered.

Why does the scale of space matter? There are obvious differences between navigating vista spaces and navigating environmental spaces. First, navigating large environmental spaces takes longer than navigating vista spaces and requires traversing through several connected (vista) spaces. It therefore requires integration of information over extended periods of time as well as space and involves the planning of complex routes which may feature a large number of decision points. Second, target locations in environmental space are beyond the sensory horizon while they lie within the sensory horizon in vista spaces. These differences have important implications for the spatial representations and the cognitive processes involved in navigation and will be discussed in more detail below.

The most commonly used navigation tasks in animal research are all vista space paradigms. The Morris Water Maze (MWM), the T-, Plus- or Y-maze are set up such that the entire environment along with all the navigationally relevant cues and the target destination can be perceived, either at all times or when making movement decisions. Accordingly, the bulk of our knowledge about the neuronal mechanisms involved in egocentric or allocentric navigation that comes from electrophysiological and behavioral neuroscience experiments in animals relates to navigation in small scale vista spaces. While environmental scale spaces are more commonly used in behavioral and brain imaging navigation experiments in humans, the question of how behavioral and neuronal mechanisms in vista and environmental scale spaces relate has received very little attention. This is somewhat surprising, given that one may argue that navigation abilities and the corresponding spatial representation have evolved to support navigation in environmental spaces where—in contrast to vista scale spaces—target destinations are not visible and cannot be reached by a simple visual approach (discussed in more detail below).

#### How do representations of vista and environmental spaces relate?

In principle, there are two ways of representing environmental scale spaces. First, the entire environment is represented in a single reference frame; depending on the nature of this reference frame, locations in the environment are either described as coordinates relative to the origin of an allocentric coordinate system, relative to other locations (allocentric), or as vectors relative to self in an egocentric reference frame. Second, different parts of the environment are represented independently; in order to navigate environments successfully, these independent representations have to be linked.

It is hard to imagine that our spatial knowledge about large environments such as entire cities is represented in a single reference frame: given an allocentric representation, where would be the origin of the coordinate system (see also discussion in Section Egocentric vs. Allocentric Reference System)? Alternatively, given a purely egocentric reference frame, one would need to constantly update egocentric vectors to all known locations in the environment during navigation. There is also empirical evidence challenging the idea that environmental spaces are represented within a single reference frame. For example, people automatically update object locations in their immediate surrounding (vista space) while more remote parts of the environment are not efficiently updated (Wang and Brockmole, 2003). Evidence for separate or fragmented representations also comes from pointing experiments, demonstrating increased accuracy for within-region as compared to between-region direction judgements (Han and Becker, 2014). This suggests that representations of environmental scale spaces are fragmented into independent units. Similar conclusions have been drawn by Meilinger et al. who demonstrated that different (vista) spaces along a route were encoded using independent local reference frames (Meilinger et al., 2014). A neuronal code for an egocentric representation of vista spaces has been hypothezied by Byrne et al. (2007): in their model, a population of posterior parietal neurons—presumably located in the precuneus—represents the locations of all landmarks and objects visible from the current location or from a location that is recalled from previous experience.

Further support for the idea of independent representations or reference frames (Worden, 1992; Derdikman and Moser, 2010) comes from animal experiments demonstrating that (i) the same place cell may code for different locations in different environments (e.g., Skaggs and McNaughton, 1998; Colgin et al., 2008); and (ii) that entorhinal grid cells do not exhibit periodic two dimensional firing fields covering the entire environments in environments that are subdivided into multiple corridors, but rather establish separate grid patterns for each corridor (Derdikman et al., 2009).

**Figure 3:**
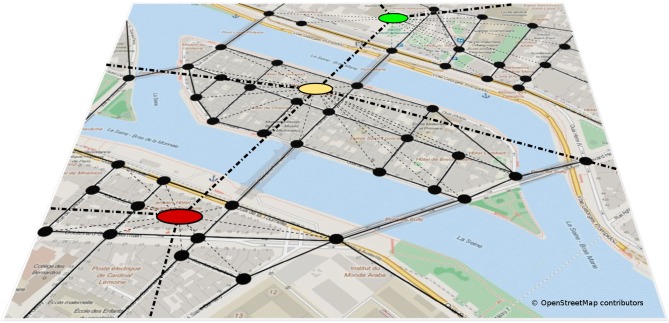
**Hierarchical graph-like representation of an environmental scale space.** Single places or vista scale spaces are represented as nodes, connections between them are represented by edges. Graphs also allow to represent hierarchical spatial knowledge, where several places are combined to form regions. The spatial relationship between different regions is represented at a higher level of abstraction (Wiener and Mallot, 2003).

How are these individual representations of smaller (vista scale) spaces connected? Graph-like representations have long been suggested to provide a structure suitable to integrate independent, yet interconnected, memories of space (Kuipers, 1978; Poucet, 1993; Schölkopf and Mallot, 1995). In addition, graphs also allow for hierarchical spatial representations (Stevens and Coupe, 1978; Wiener and Mallot, 2003; Han and Becker, 2014). In graph-like structures, local positional information is usually represented in nodes, while edges represent connections between nodes (see Figure 3). Several graph models of environmental scale spatial memory have been proposed in the animal and human literature. The exact nature of the information stored in nodes and edges differ between models: Poucet (1993), for example, suggested that nodes are place representations, while connections between distinct places are encoded in polar coordinates as vectors. At a higher level of abstraction, the animal’s current environment, which may contain any number of places that share common stimulus properties, becomes a so-called local chart. Note that the idea of a local chart is similar, if not identical, to the concept of a vista space. According to Poucet (1993) environmental scale spaces are represented in terms of multiple local charts (vista spaces) and spatial relationships between them. Closely related to this network of charts idea is the *Network of Reference Frames* theory (Meilinger, 2008), which proposes that environmental spaces are represented by means of interconnected reference frames, i.e., independent coordinate systems each with a specific orientation and representing a different vista space. These reference frames or nodes are connected by edges which describe the perspective shift—the translation and rotation—necessary to move between them.

## What do we know about the neural mechanisms that support navigational behavior?

### Navigation in vista scale spaces

#### Allocentric navigation in vista space

Ever since the discovery of place cells in the hippocampus of freely moving rats (O’Keefe and Dostrovsky, 1971), the hippocampus has been thought to provide an allocentric description of an environment, often referred to as a cognitive map. Place cells fire whenever an animal moves through a certain location, independent of its facing direction. Many experiments have shown that both environmental boundaries and salient landmarks placed outside the apparatus exert tight control over the firing of place cells, suggesting that place cells are driven by environmental objects located (i) in a certain allocentric direction from the animal; and (ii) at a certain distance from the animal. This distance sensitivity relative to the observer, however, makes it impossible to determine whether place cells code for distance in an egocentric or an allocentric reference frame, since both are identical when the observer occupies one of the two points between which distance is computed.

**Figure 4:**
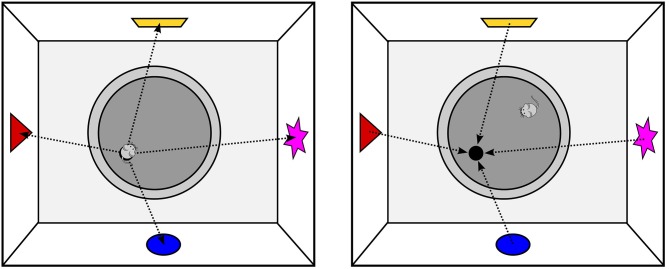
**Spatial Coding in the Morris Water Maze;** **left:** the animal has found the platform and can encode the spatial relationship between the platform and extra-maze cues as allocentric vectors. Note that while sitting on the platform, allocentric and egocentric vectors to the landmarks only differ with respect to their orientation, but not in their length; **right:** as the entire environment can be perceived from any position in the pool, the platform location can be computed by simply projecting the learned allocentric vectors from the landmarks into the pool. As a consequence, the animal does not need to know its current allocentric position to find the submerged platform.

Electrophysiological recordings of place cells, however, can only demonstrate a correlation between spatial position and the firing behavior of neurons. To establish the causal role of the hippocampus for allocentric navigation, numerous studies have applied temporary or permanent lesions to the hippocampus. By far the most popular paradigm used in these experiments is the MWM (Figure 4), which is often referred to as the gold standard for studying allocentric spatial learning or memory. In the MWM, the animal has to find a platform submerged in a pool of opaque water, a task that healthy animals learn quickly even though the starting location is changed between trials or sessions. In contrast, animals with hippocampal lesions show marked impairments in the MWM as they take longer to learn the location and tend to often search for the platform in incorrect locations (Morris et al., 1982). However, when these animals always begin the trial from the same position, they show little to no impairment. Taken together, these findings suggest that the hippocampus contributes only to allocentric spatial learning, whereas egocentric navigation is independent of the hippocampus.

While the results from these studies seem to tell a coherent story about the navigational functions of the hippocampus, a closer look reveals a number of problems and limitations. In the MWM, the location of the goal—i.e., the position of the hidden platform—can either be specified by an allocentric vector that specifies the distance and direction from the origin of a coordinate system or by multiple allocentric vectors between the platform location and other objects in the environment. As discussed above, it is unclear what would specify the origin of an overall allocentric coordinate system in the MWM, hence it seems far more likely that the allocentric representation involves vectors between the platform location and external landmarks.

What is rarely appreciated is that the MWM only assesses navigation in vista space. Specifically, no matter where the animal is started on a given trial, the entire environment can always be perceived with little or no movement (note that rodents have a very large field of vision; even with a more restricted field of vision, head movements are sufficient to perceive the entire environment). In other words, all information required to calculate the platform location is available from the animals current location, and the platform location can therefore be computed by simply projecting the allocentric vectors from the landmarks into the pool (see Figure 4). As a consequence, the animal does not need to know where it is located in the environment, since the platform location can easily be computed by looking at the landmarks. This *lack of the need for self-localization* also applies to other popular vista space navigation tasks. For example, in the T-, Y- or Plus Maze paradigms, the entire space is visible from the central junction, hence direct approach behavior to the goal without self-localization is possible.

Finally, and contrary to widely held assumptions, it is not easy to identify the spatial reference frame used in vista space navigation tasks. For example, in the control condition used in many MWM studies, rats simply have to approach a visible platform. Animals with hippocampal lesions perform well in this version of the task, which is typically taken as evidence that the hippocampus is specifically required for allocentric spatial memory. However, this control condition does not require any spatial learning, because the goal is always visible. Similarly, studies with humans with hippocampal lesions have employed egocentric control conditions in which the platform location is marked by a nearby beacon (Goodrich-Hunsaker et al., 2010), which does not require the learning of an egocentric vector either. As a consequence, the only conclusion that can be drawn from such studies is that the hippocampus plays a role in forming spatial memories, but it does not inform about the nature of the reference system. To overcome this problem, some authors have employed control conditions in which the platform continues to be submerged but the animal is always started from the same location (Eichenbaum et al., 1990), assuming that it now solves the task by learning an egocentric vector from its starting location towards the goal. Even this control condition is problematic because (i) it is now unclear what information is used to reference the goal location, because both extramaze landmarks and the animal’s starting position could serve this purpose; and (ii) it is possible that the task is generally easier when the animal is always released from the same starting location. A solution to this problem would involve a condition in which the animal is always started from a different location—as in the allocentric condition—but the platform is moved so that it remains in a constant egocentric relation to the animal’s starting position. With this manipulation, both egocentric and allocentric conditions would require the animal to learn vectors, either relative to external room cues (allocentric) or relative to its own position (egocentric).

While our discussion of allocentric navigation in vista space has focussed on studies investigating spatial coding in the hippocampus, the discovery of grid cells in entorhinal cortex (EC) of rodents and primates shows that an allocentric code for an organism’s position may already be computed upstream of the hippocampus (Hafting et al., 2005; Killian et al., 2012; Jacobs et al., 2013). While theoretical models assume that the main function of the grid cell system consists of path integration—the tracking of changes in spatial position based on incoming self-motion cues—most studies on spatial coding in EC have employed electrophysiological recordings in freely moving animals that were not engaged in any navigational task. In addition, lesions studies investigating the role of EC for allocentric learning (i.e., in the MWM) have yielded mixed results (Burwell et al., 2004; Steffenach et al., 2005). Given these findings and the interpretational problems of the MWM and other vista space navigation tasks as discussed above, it is difficult to ascertain the navigational contribution of the grid cell system to vista space navigation at present.

#### The role of boundaries and geometric layout

In rectangular arenas, rats often confuse diagonally opposite corners, even when differentiated by distinctive cues (Cheng, 1986). This led to the claim that rats rely preferentially on the geometry of space, encoded in a dedicated geometric module. Similar ideas have been proposed for humans, even though they may combine geometric cues with featural cues (i.e., landmarks) by means of natural language (Wang and Spelke, 2002). While later studies have cast doubt on the idea of a dedicated geometric module in the mammalian brain (Cheng, 2008), behavioral work has consistently shown that salient geometric cues such as the walls of a room are often used to define allocentric reference directions (Shelton and Mcnamara, 2001).

On the neuronal level, two cell types have been described that may provide a neural substrate for the coding of geometric layout. Border cells fire whenever the animal is at an environmental boundary such as a wall of the recording arena (Solstad et al., 2008). In addition, boundary vector cells, which have been found in the subiculum, fire whenever the animal is located in a certain distance and allocentric direction away from a boundary (Lever et al., 2009). Importantly, given that hippocampal place cells appear to function independently of the grid cell system (Brandon et al., 2014), these boundary vector cells may provide the key input that drives the localized firing of hippocampal place cells (Bush et al., 2014).

In the context of our discussion of vista and environmental space, it is important to note that virtually all behavioral and neuroscience experiments on geometry coding have been conducted in vista space environments such as single rooms for humans or regularly shaped boxes (i.e., rectangles) for rodents. In these environments, salient geometric boundaries that define the layout of a space can be seen from any vantage point, hence it makes perfect sense that distance and direction to the boundaries are (i) explicitly coded in neuronal circuits; and (ii) used to organize spatial representations and to reference the locations of external objects and of the navigator. A major problem, however, arises when we need to code for a location that lies beyond our sensory horizon: in environmental spaces such as neighborhoods or different parts of a city, geometric cues that could be seen from any vantage point are rarely available (note, of course, that there are exceptions such as the extended cliff face of Edinburgh’s famous Salisbury Crags that, due their elevated location, are visible from most parts of the city). Rather, all one can see when wandering on a typical street or square are the faces of the buildings in the local surroundings, which disappear quickly upon turning into another street. Geometric cues such as rivers or extended major roads, which could be useful for coding locations in large scale space, can often not be seen or, even if they are within one’s current sensory horizon, only parts of them are visible. As a consequence, environmental boundaries and boundary related coding appear most useful for coding local positions in vista spaces such as the location of a traffic light relative to the surrounding buildings, but they are rarely available and useful in environmental scale spaces.

#### Egocentric navigation in vista space

Similar to allocentric navigation, animal studies on navigation involving egocentric knowledge are predominantly carried out in vista space environments. For example, in the MWM, lesions to the striatum impair navigation towards a location marked by a distinct visible landmark but not to an unmarked one defined relative to distal landmarks and boundaries (Packard and Mcgaugh, 1992; McDonald and White, 1994). Similarly, when a location is defined by its distance and direction from an intramaze landmark (given distal orienting cues), and not by the boundary of the maze, hippocampal damage does not impair navigation (Pearce et al., 1998) although lesions of the anterior thalamus (with presumed disruption of the head-direction system) do impair navigation (Wilton et al., 2001). These findings, which are in accord with the results from fMRI experiments in humans (Doeller et al., 2008), are generally taken as evidence for the complementary roles of the hippocampus and the striatum for spatial navigation, with the former defining locations relative to the boundaries and extramaze landmarks in an allocentric reference frame, while the latter defines locations relative to local landmarks, and the head direction system is required to derive the animal’s orientation from distant landmarks. A second type of studies have consistently implicated the dorsal striatum, in particular the caudate, in learning habits and egocentrically defined motor responses. For example, in the Plus Maze task, the response strategy consists of always executing the same motor response at the central junction (i.e., turn right), independent of the direction from which this junction is approached. Inactivating the caudate leads to a blocking of response learning and a preference for a place strategy, whereas inactivating the hippocampus has the opposite effect (Packard and Mcgaugh, 1992).

These complementary roles of the dorsal striatum: (i) coding for object positions relative to local landmarks or beacons; and (ii) defining an egocentric motor response, however, do not tap into the process of coding egocentric knowledge as defined in Figure 1. Rather, coding for object positions relative to local landmarks involves an observer independent object-to-object vector that requires the retrieval of an allocentric reference direction. In addition, learning to execute a specific motor response such as “turn right” does not involve the learning of a metric egocentric vector between the observer and a target object. All that is needed is motor skill or habit learning in which a categorical motor behavior such as “turn right”, which is not specified in terms of a precise angle, is associated with obtaining a reward. In support of this conceptualization, response learning in the Plus- or T-maze is known to proceed slower than place learning (Packard and Mcgaugh, 1992), supporting the notion that response learning requires repeated reinforcement whereas place learning is a form of rapid, incidental learning (Salmon and Butters, 1995).

Direct evidence for the coding of egocentric vectors towards external objects in vista space comes from primate studies investigating the functions of the posterior parietal cortex. When monkeys are presented with a visual or auditory target, neurons in subdivisions of the posterior parietal cortex code for the object’s location in multiple egocentric reference frames, for example eye-centered, head-centered or trunk-centered (Colby, 1998). Such locational cues form the basis of an egocentric map of the surrounding space that critically depends on the precuneus and connected inferior and superior parietal areas. Similar egocentric representations have been documented in humans using fMRI (Committeri et al., 2004; Schindler and Bartels, 2013), but it is important to note that these studies are generally performed with static observers. Egocentric navigation, however, involves a dynamic updating of the egocentric object vectors as the observer moves about an environment. This integration of self-motion cues with existing egocentric representations appears to be mediated by the precuneus (Wolbers et al., 2008; Jahn et al., 2012). It is currently not clear, however, as to whether the precuneus also provides egocentric codes for distant objects beyond the sensory horizon—a key component of navigation in environmental scale space. In addition, whether the role of the precuneus is restricted to providing transient egocentric codes or whether it also stores long-term memory traces of such codes is unknown.

### Navigation in environmental scale spaces

Navigation in environmental scale spaces can be based on allocentric or egocentric strategies (Wiener et al., 2013). Functional brain imaging studies in humans have shown that the hippocampal circuit is recruited when people employ strategies that require allocentric processing, such as planning and navigating novel routes through familiar environments (“wayfinding”). The parietal cortex and striatal circuits, in contrast, are involved in egocentric navigation strategies such as following a well-known route (Hartley et al., 2003; Iaria et al., 2003; Wolbers et al., 2004; Burgess, 2008). While these results seem to mirror the findings of lesion and electrophysiological studies in animals in vista scale space nicely (discussed above), it is important to consider the impact that the scale of space has on the mechanisms involved in navigation.

#### Allocentric navigation in environmental spaces

Wayfinding is the most commonly used allocentric navigation task in environmental spaces (e.g., Wiener et al., 2004; Hölscher et al., 2011). Wayfinding in familiar environments such as cities, neighborhoods or buildings involves planning novel routes to destinations beyond the current sensory horizon (i.e., in a different vista space). During wayfinding, landmarks specifying the goal location will not be available at the start of the navigation. Thus, in contrast to allocentric navigation in vista spaces, the goal location cannot be computed simply by looking at the landmarks. Rather, computing a trajectory to the goal location requires several steps. The first step involves localizing oneself within the environment, a process that, as we discussed before, is not required in vista scale space navigation. In a second step, the goal needs to be localized. These first two steps can be conceptualized as highlighting two locations in an internal representation of space or cognitive map. Finally, the actual trajectory from the current location to the goal has to be computed. Importantly, this trajectory will rarely be a straight line as it is in tasks such as the MWM. Instead, navigation in environmental spaces often requires planning and following long paths with several decision points. Simply choosing the path option most aligned with the direction of the goal location does not necessarily lead to the destination. This has important implications for the underlying internal mental map; representations only coding spatial relationships between landmarks or locations are not sufficient. Wayfinding in environmental spaces also requires knowledge about connections between places which is difficult—if not impossible—to accomplish if all spatial knowledge is coded in a single allocentric reference frame (i.e., in map like representations of space). As mentioned in Section Scales of Space, the simplest structure to achieve this is a graph-like representation, in which nodes represent places (or vista spaces) and edges refer to the movements required to navigate between neighboring nodes.

In humans, functional brain imaging studies suggest that the hippocampus is recruited during the learning of environmental spaces (cognitive mapping) and when planning routes or shortcuts through known environments: Wolbers and Büchel (2005) trained participants on a long and complex route featuring several places and landmarks and asked participants to judge the spatial relations between these landmarks. Interestingly, hippocampal activation did not follow overall performance but rather reflected the amount of knowledge acquired in a given experimental session, suggesting prominent hippocampal activation only during early stages of cognitive mapping. Furthermore, hippocampal activation is associated with planning routes through spatial but not social (purely relational) networks (Kumaran and Maguire, 2005) as well as with the quality of the solution (Hartley et al., 2003).

Although these studies demonstrate that the hippocampus is involved in allocentric navigation in environmental spaces, they do not reveal its precise role during wayfinding. As discussed above, wayfinding requires localization of self and destination and the planning of a route between these locations. Furthermore, the route needs to be monitored during travel and further planning en route is often required (Wiener and Mallot, 2003; Hölscher et al., 2011). To isolate the neuronal mechanisms underlying these processes, Spiers and Maguire had experienced London Taxi drivers navigate through a virtual reality simulation of London (Spiers and Maguire, 2007a,b). Using retrospective verbal protocols, they isolated different cognitive processes involved in wayfinding and related them to functional brain imaging data recorded during the actual navigation. Neural activity associated with route planning was not limited to the hippocampus, but involved a network of different regions including retrosplenial cortex and prefrontal cortex (PFC). Specifically, hippocampal activation was most prominent during initial planning, suggesting its primary role is to activate or retrieve the relevant spatial information, and the retrosplenial cortex was most active during planning and monitoring progress along the route. In addition, PFC was involved in planning, monitoring, when expectations are violated or when encountering unexpected roadblocks which required replanning of the route. Finally, during navigation, the spatial relationship between self and goal location was continuously tracked by several brain regions. Medial prefrontal activity correlated positively with beeline distance to the target; activity in right subicular/enthorinal region correlated negatively with target distance, and the posterior parietal cortex coded for the egocentric direction to the target. The latter result is in line with findings obtained in vista space paradigms, demonstrating that the posterior parietal cortex represents spatial location relative to a number of egocentric reference frames (Colby, 1998, see also Section “Egocentric Navigation in Vista Space”).

Taken together, successful allocentric navigation in environmental space involves a number of processes, including self localization, the planning of complex routes, monitoring progress along the route and further (re)planning, that are not necessary when navigating vista spaces. As a consequence, research using vista scale paradigms such as the MWM, the Plus- or T-maze will only be able to identify a subset of the neuronal mechanisms involved in environmental space navigation.

#### Learning unfamiliar environments

Another important difference between navigation in vista and environmental scale spaces relates to the learning of the environment. To solve the allocentric version of the MWM task, for example, subjects have to know the position of the hidden platform relative to the environmental cues. Essentially this information can be encoded after finding the platform either by taking a snapshot of the sensory information which can be compared to the sensory input during navigation to calculate goal direction (Cartwright and Collet, 1983; Cheung et al., 2008), or by memorizing the spatial relations (i.e., vectors) between self-location and environmental landmarks. Learning environmental scale spaces is different as information about the entire environment cannot be acquired instantaneously, but is experienced over an extended period of time during exploration. That is, information about different parts of the environment is experienced at different times and has to be combined into an integrated representation of space. Knowledge about spatial relations between different parts of the environment comes from fundamental navigation mechanisms such as path integration and spatial updating which inform about the translation and rotations when navigating between places.

#### Egocentric navigation in environmental spaces

The prototypical egocentric navigation task in environmental spaces is route navigation or route following. In humans, route knowledge is often conceptualized as a series of stimulus-response (S-R) associations (Trullier et al., 1997): the recognition of a place or landmark triggers a movement response that is coded in an egocentric reference frame. In the so-called associative cue strategy this response is explicitly directional (e.g., “Turn left at the Gas Station”). In contrast, in the beacon strategy, the response requires a movement or turn towards a landmark or beacon (e.g., “Turn towards the Gas Station”). Finally, route navigation can also be supported by simply memorizing a series of direction changes (“Left—Right—Right - …”, Waller and Lippa, 2007), in particular when the environment does not provide any salient landmarks.

Route learning studies in both real and virtual environmental scale spaces demonstrated that objects located at decision points are more reliably remembered than those that were located between decision points (Aginsky et al., 1997; Schinazi and Epstein, 2010). When presented with isolated objects that were encountered during route learning, neuronal activity in the parahippocampal gyrus is modulated by the navigational relevance of these objects, with strongest activation for objects at decision points that serve as landmarks or associative cues for route knowledge (Janzen and van Turennout, 2004; Janzen and Weststeijn, 2007; Schinazi and Epstein, 2010). Moreover, both behavioral and neural responses to the landmark-object are modulated when primed by an adjacent landmark. Specifically, faster behavioral responses and increased activation in the retrosplenial complex (RSC) are associated with in-route vs. against-route primes, suggesting that the RSC is involved in integrating landmark and path information (Schinazi and Epstein, 2010). These findings are in line with earlier studies, suggesting that retrosplenial areas play an important role in the learning of spatial relationships in large environmental spaces (Wolbers and Büchel, 2005). Electrophysiological findings in monkeys, demonstrating that neurons in the medial parietal regions, analogous to the human RSC, selectively respond to specific S-R associations, are consistent with the idea that the RSC supports associative cue based route learning (Sato et al., 2006).

Some early evidence using standard vista space paradigms such as the T-maze suggest that allocentric strategies can be adopted faster than egocentric response strategies (Tolman et al., 1946) In environmental space, in contrast, parallel acquisition of egocentric and allocentric strategies has been shown in both animals (Rondi-Reig et al., 2006) and humans (Iglói et al., 2009). It is likely that differences in the strategy adopted between vista and environmental spaces result from differences in egocentric navigation strategies. Specifically, learning an egocentric response strategies in vista space paradigms such as the T- or Plus-Maze involves only a single response that is best described as a striatum-dependent motor-skill or as habit learning which involves repeated reinforcement (Salmon and Butters, 1995). Egocentric navigation in environmental space, in contrast, usually involve associative components (“Turn right at the church”) as well as memory of a series of movement decisions, the execution of which takes much longer than a single vista space response. The learning of habitual motor programmes that support egocentric navigation in vista spaces may therefore be less suited for environmental scale space.

In contrast to the widely held view that the hippocampus is involved in allocentric but not egocentric processing during navigation (Byrne et al., 2007; Whitlock et al., 2008; Banta Lavenex and Lavenex, 2009), a number of studies have implicated the hippocampus in route learning. Barrash et al. (2000), for example, demonstrated that patients with hippocampal lesions showed impaired route learning performance. Marked route learning impairments have also been demonstrated in patients with Mild Cognitive Impairment (MCI) and early stage Alzheimer’s Disease (Cushman et al., 2008; Pengas et al., 2010), which are primarily characterized by neurodegenerative changes in the medial temporal lobe (Whitwell et al., 2007). Furthermore, knock out mice lacking hippocampal CA1 NMDA receptors are not only impaired in allocentric learning but also in learning a sequence of self-movements (sequential-egocentric learning: “left-right-left”; Rondi-Reig et al., 2006). Interestingly, these knock out mice were not impaired in learning a single self-movement (“turn left”) as required for T- or Plus-Maze vista space paradigms, but only showed deficits in learning successive body turns. These results are in line with findings implicating the hippocampus in the learning of complex motor sequences (Schendan et al., 2003; Gheysen et al., 2010).

Another explanation for the involvement of the hippocampus in route learning comes from its role in episodic memory (Eldridge et al., 2000; Burgess et al., 2002), because each travel through an environment also represents an episode during which different places (or vista spaces) are sequentially experienced. Remembering such a route or episode requires associating places that one has occupied with movement decisions. Indeed, evidence from electrophysiological studies suggest that the hippocampal code for positional information is modulated by past experiences. For example, when rats navigate along more complex paths or routes, firing rates of hippocampal place cells may vary in the same location depending on where the animal has come from (Frank et al., 2000; Wood et al., 2000).

## Conclusion/summary

In this review we have highlighted a number of problems arising in the study of the neuronal mechanisms supporting egocentric and allocentric reference frames in navigation. First, and in contrast to egocentric reference frames, the nature of allocentric reference frames is somewhat ill defined. For example, any allocentric reference frame requires an origin for its coordinate system. In most environments, however, it is unclear where this origin would lie and whether locations are encoded by means of allocentric vectors relative to the origin of the coordinate system or by their spatial relations to other locations. While some of this confusion may be related to the term “allocentric” itself which implies that the reference frame has a center, it results in different usage of the term in the literature and the exact nature of the assumed allocentric reference system often remains underspecified.

Second, to study the neuronal mechanisms involved in allocentric processing one ideally wants a purely allocentric navigation task. However, all navigation paradigms have egocentric components, as actual navigation requires information—such as direction to the destination or direction of turn—in an egocentric format when planning and executing body movements. In allocentric navigation paradigms such as the MWM it is not easy to clearly characterize the allocentric and egocentric components, which makes it more difficult to allocate neuronal activity to these different reference frames.

Third, the egocentric conditions used in the prominent vista space paradigms do not require the processing of egocentric vectors and may therefore not constitute the best possible comparison to hippocampal-dependent allocentric conditions for which the processing of object-to-object vectors is required. In egocentric vista space navigation subjects need to associate beacons with platform positions (MWM) or learn a motor response (Plus Maze). The egocentric information that is learned in these situation is categorical (next to, left, right) rather than metric (vector) and solving these tasks involves habit or motor skill learning to a much greater extend than allocentric versions of the task.

Fourth, ultimately navigation is about reaching destinations beyond the current sensory horizon, that is, it is about movement in environmental space. The vast majority of animal studies addressing the neuronal basis of navigation, however, have employed small scale vista space paradigms. Our knowledge about how results from these studies translate to navigation in environmental scale spaces is limited. We have argued that successful navigation in environmental space, both egocentric and allocentric, involves a number of processes that are not required in vista space navigation. Research using vista scale paradigms will therefore only be able to identify a subset of the neuronal mechanisms involved in everyday navigation. This is demonstrated in findings implicating the hippocampus in route learning in environmental scale spaces—the prototypical egocentric task in environmental spaces. Such findings challenge the notion that the hippocampal circuit is recruited exclusively for allocentric navigation while the striatal circuit is responsible for egocentric navigation and demonstrate that future work needs to address the relationship between neuronal mechanisms underlying navigation in vista and environmental spaces.

## Conflict of interest statement

The authors declare that the research was conducted in the absence of any commercial or financial relationships that could be construed as a potential conflict of interest.
